# Chloroplast genome assembly, annotation, comparative genomics, and genetic diversity analysis of a vulnerable endemic species *Lavandula maroccana* Murb

**DOI:** 10.1371/journal.pone.0345166

**Published:** 2026-03-31

**Authors:** Manosh Kumar Biswas, Jean Legeay, Khaoula Errafii, Imane Abbad, Pat Heslop-Harrison, Mohamed Hijri, Bulbul Ahmed

**Affiliations:** 1 African Genome Center (AGC), University Mohammed VI Polytechnic, Benguerir, Morocco; 2 Department of Genetics and Genome Biology, Institute for Environmental Futures, University of Leicester, Leicester, United Kingdom; 3 Faculty of Sciences Semlalia, Cadi Ayyad University, Marrakech, Morocco; 4 Institut de Recherche en Biologie Végétale, Département de Sciences Biologiques, Université de Montréal, Rue Sherbrooke Est, Montréal, Quebec, Canada; Central University of Punjab, INDIA

## Abstract

*Lavandula maroccana* Murb., an endemic species of the western Mediterranean, is classified as vulnerable and was added to the International Union for Conservation of Nature (IUCN) Red List in 2020. We assembled and annotated the chloroplast genome and compared it with chloroplast genomes of five previously published *Lavandula* species. The assembled chloroplast genome of *L. maroccana*, spans 151,323 bp, and exhibits a typical quadripartite structure, consisting of an 82,861 bp large single-copy (LSC) region, a 17,452 bp small single-copy (SSC) region, and two 25,505 bp inverted repeat (IR) regions. The genome has a GC content of 38% and contains 110 unique genes, 37 tRNA genes, and 4 rRNA genes. A total of 37 perfect SSR motifs were identified, with most being mononucleotide repeats. Pentanucleotide and hexanucleotide repeats were absent as perfect motifs but were present as imperfect motifs at a lower frequency than other repeat types. AT-rich SSRs were more prevalent than GC-rich motifs. Palindromic and forward repeats were more frequent than complementary and reverse repeats. Nucleotide sequence diversity (Pi) values ranged from 0.004 (*atpH*) to 0.035 (*matK*), with protein-coding genes found to be under purifying selection. Phylogenomic analyses, based on the complete plastome sequences and the *matK* + *trnL* genes, revealed that intra- and inter-species diversity within *Lavandula* species. We compared the genomes, and their evolutionary relationships, and genetic structures with published chloroplast genomes from other species- *L. maroccana*, *L. angustifolia*, and *L. dentata*.

## Introduction

*Lavandula maroccana* Murb., commonly known as Moroccan lavender, belongs to the genus *Lavandula* within the family Lamiaceae, and order Lamiales. The genus *Lavandula,* comprising approximately 47 species of perennial flowering plants [1], is further categorized into three subgenera and eight sections based on morphological characteristics [2]. *Lavandula* species are predominantly distributed across drier and warmer regions of the world, with their native range spanning the Mediterranean Basin, including Southern Europe and North Africa, as well as parts of Eastern and Southern Africa, the Middle East, and South Asia [3]. In Morocco, nine *Lavandula* species have been identified, five of which are endemic [4]. *L. maroccana* is native to the western Mediterranean and endemic to the High Atlas of Morocco. However, it is also found across three floristic divisions in the High, Anti-, and Middle Atlas regions of Atlantic Morocco [5]. Morphologically, Moroccan *Lavandula* species resemble other Mediterranean lavenders, such as *Lavandula angustifolia* and *Lavandula stoechas*. However, they exhibit distinct adaptive traits that enable them to thrive in Morocco’s diverse landscapes, particularly in rocky soils and at higher altitudes.

Lavender is rich in bioactive compounds such as linalool and linalyl acetate, which contribute to its aromatic properties. Compounds like 1,8-cineole, camphor, and beta-caryophyllene provide anti-inflammatory and antimicrobial effects, while terpinen-4-ol and geraniol offer antiseptic and antioxidant benefits. Additionally, coumarins and tannins contribute to its healing and antifungal properties. The chemical composition of lavender varies depending on the species and growing conditions. Lavender is well-known for its essential oils and widely used in perfumes, skincare products, and traditional medicines [6–10]. Consequently, lavender holds significant economic importance, particularly in the cosmetics, pharmaceutical, and aromatherapy industries. Moroccan lavender, in particular, is prized for its high-quality essential oils. In Morocco, lavenders have been traditionally used to treat various health conditions, including digestive, respiratory, gastrointestinal, nervous, and inflammatory disorders. They are also used in herbal teas, traditional meals, and cosmetics. Dried lavender flowers are often placed under pillows to promote sleep and relaxation [11].

*L. maroccana* faces several threats primarily due to overharvesting and unsustainable practice harvesting practices. Local communities often cut entire plants, including their roots, for domestic purposes such as fuel, medicinal uses, and traditional remedies. This practice not only removes the plants but also hinders their natural regeneration, further depleting the population. In addition to overharvesting, deforestation and climate changes pose significant challenge, as they reduce the natural habitat available for *L. maroccana* to grow and thrive. These combined threats present a complex challenge to the survival of *L. maroccana*, emphasizing the urgent need for conservation efforts and sustainable management practices to protect this vulnerable species [5].

Chloroplasts are essential organelles in plants, responsible for photosynthesis and various metabolic functions. They contain their own distinct genome, which is maternally inherited, smaller in size, and less prone to recombination compared to nuclear genomes. Chloroplast (cp) genomes are highly conserved, with specific variable regions, such as the *ycf4-cemA* fragment, serving as molecular markers for distinguishing closely related species [12]. A typical cp genome consists of a large single-copy (LSC) region, a small single-copy (SSC) region, and two inverted repeats (IRa and IRb) [13,14], which contribute to its stability and make it highly valuable for phylogenetic studies. Understanding the cp genome in the context of overall evolutionary dynamics can elucidate the phylogenetic relationships among species within the family. Sequencing, assembling and annotating the complete cp genome of *Lavandula* species offers critical insights into species evolution, genetic diversity, and interspecies relationships within the genus. Advances in high-throughput sequencing technologies have enabled the acquisition of complete chloroplast genome sequences from whole-genome data, successfully applied to plant species, including *Phoenix dactylifera* [15], *Taraxacum spp.* [16], *Avena sativa* [17], *Phalaenopsis* spp. [18], *Zingiber* spp. [19], *Ligustrum* spp. [20], *Aconitum transsectum* [21], *Cucumis melo* [22], *Tuberculata* spp. [23], *Vernicia spp.* [24], *Amorphophallus spp.* [25], and *Pseudogalium* [26].

To date, only the complete chloroplast genomes of *L. angustifolia* and *L. dentata* have been sequenced, assembled and annotated, with their sequences publicly available in the NCBI database. Currently, no genome sequences or sequence data exist for Moroccan lavender species. The available cp genomes can serve as valuable resources and references for comparative chloroplast genomics of *Lavandula* species, as well as for developing molecular markers. In this study, we sequenced, assembled the complete chloroplast genome of *Lavandula maroccana* using Illumina NextSeq instrument and compared it with other *Lavandula* species to facilitate comparative genomics, SSR analysis, and marker development. We analyzed the cp genomic structures of *L. maroccana*, *L. angustifolia*, and *L. dentata*, conducting comparative analyses of their genomes, evolutionary relationships, and genetic structures alongside published chloroplast genomes from other species in the Lamiaceae family. This study establishes a foundation for future research in plastidomics, genetic evolution, and the precise molecular identification of *Lavandula* species.

## Materials and methods

### Plant material, DNA extraction and sequencing

Shoot samples of *Lavandula maroccana* were collected from Ijoukak region in Morocco (31°00’N and 8°09’W, 1267 m). The plant was identified by Imane Abbad, one of the co-author of the manuscript, and voucher specimen (LM-34) have been deposited at the Laboratory of Microbial Biotechnologies at the Agrosciences and Environment of the Faculty of Science Semlalia, University of Cadi Ayyad, Marrakech, Morocco. Since the plant was collected in non protected area, no license or legal permission was required. High molecular weight total genomic DNA was extracted from the 100 mg of fresh leaves using DNeasy Plant Pro Kits (Qiagen, Global Diagnostic Distribution, Temara, Morocco). DNA concentration and quality were measured using Qubit™ 4 Fluorometer (Thermo Fisher Scientific), and quality was verified by electrophoresis on a 1% (w/v) agarose gel and Femto Pulse (Agilent Technologies). Library preparation was performed using the Illumina DNA Prep Kit (Illumina, MegaFlex, Casablanca, Morocco) according to the manufacturer’s instructions with slight optimizations. DNA was tagmented with bead-linked transposes to an average size of ~550 bp, followed by end repair, A-tailing, and ligation of dual-index adapters. The ligated DNA was purified using AMPure XP beads (Beckman Coulter, Fisher Scientific, Master Lab, Rabat, Morocco) and amplified by PCR to enrich the library. The quality and size distribution of the prepared libraries were confirmed using an Agilent Bioanalyzer 2100 (Agilent Technologies). The sequencing run was executed using the Illumina NextSeq 550 system (Illumina, MegaFlex, Casablanca, Morocco). Libraries were loaded onto the flow cell and sequenced using a 300-cycle High-Output Kit for paired-end reads. The sequencing workflow was managed using the Illumina Local Run Manager software, which enabled real-time tracking of sequencing metrics and ensured data quality throughout the run. Upon completion, the Local Run Manager software was used for demultiplexing and generation of FASTQ files. The resulting data, with a yield of approximately 40 Gb per sample, were suitable for comprehensive downstream genomic analysis.

### Genome assembly, annotation, and sequence analyses

The raw Illumina sequence data were assessed using FastQC v.0.11.9 [27] (http://www.bioinformatics.babraham.ac.uk/projects/fastqc/) and filtered with Trimmomatic v.0.39 [28] using default parameters. Clean reads were then mapped aginest all available refences chloroplast genome from the NCBI chloroplast genome database using BOWTIE2 [29] with default parameters. Maped chloroplast reads were extracted using SAMtools v1.15 [30]. Then the extracted hiquality chloroplast reads were assembled in to the complete chloroplast genomes using NOVOPlasty v.4.0 [31] with default paraments. The assembled chloroplast genomes were annotated using GeSeq [32] an updated and widely used tool for organellar genome annotations. Additionally, tRNAscan-SE v.2.0.5 [33] and BLAST v.2.13.0 [34] were employed to confirm the tRNA and rRNA genes. The physical maps of the complete chloroplast genomes were generated using Organellar Genome DRAW (OGDRAW) v.1.3.1 [35]. The annotation results were further validated, formatted using Sequin v.15.50 from NCBI. The assembled chloroplast genome data are available under PX569468 in NCBI.

### Simple sequence repeats and repetitive DNA analysis

Simple sequence repeats (SSRs) in the chloroplast genomes of various *Lavandula* species were identified using both 3GMAT pipeline (https://github.com/mkbcit/3GMAT) and PHOBOS tool [36], to ensure comprehensive detection. 3GMAT was used to detect perfect SSRs with minimum thresholds set at 10, 5, 4, 3, 3, and 3 for mono-, di-, tri-, tetra-, penta-, and hexanucleotide repeats, respectively. PHOBOS, which can identify both perfect and imperfect SSR motifs, was used with default parameters. SSRs identified by PHOBOS were subsequently filtered based on repeat unit lengths: mono-repeats ≥10 bp, di-repeats ≥10 bp, tri-repeats ≥12 bp, tetra-repeats ≥12 bp, penta-repeats ≥15 bp, and hexa-repeats ≥18 bp. The results from PHOBOS were then compared with those obtained from 3GMAT to provide a comprehensive SSR profile. SSR markers were also developed using the 3GMAT pipeline. Other repeat sequences, such as palindromic, reverse, forward, and complement repeats in Lavandula species, were identified using REPuter v1.0 software (https://bibiserv.cebitec.uni-bielefeld.de/reputer) [37], following the parameters of a Hamming distance of 3, a maximum repeat length of 5,000 bp, and a minimum repeat size of 30 bp.

### Sequence divergence and comparative genome analysis

The expansion and contraction of the IR (inverted repeat) regions were analyzed by comparing the SC/IR borders and their adjacent genes across six complete Lavandula chloroplast genomes using IRscope [38]. Interspecific rearrangements were assessed by Mauve alignment in Geneious using the default parameters with six lavender chloroplast genomes. The divergence among the six chloroplast genomes was examined using the Shuffle-LAGAN mode of the mVISTA v2.0 (http://genome.lbl.gov/vista/index.shtml) [39], with the annotated chloroplast genome of *L. dentata* serving as the reference. Nucleotide variability (Pi) values were calculated using a pipeline implemented in Python scripts (https://github.com/Xwb7533/CPStools/tree/main). Protein-coding regions and intergenic (non-coding) regions of *L. maroccana* and the reference *L. dentata* were extracted from chloroplast genome annotation files in GenBank (.gb) format using Python scripts. These regions were then aligned using MAFFT with default parameters. The Pi values were subsequently computed using Python scripts.

### Positive selection analysis

Selective pressure was assessed through the Ka/Ks ratio, where nonsynonymous (dN) and synonymous (dS) substitution rates were calculated for protein-coding genes of the *L. maroccana* chloroplast genome using the Ka_Ks Calculator v3 [40].

### Codon usage bias analysis

Codon usage was analyzed using Python scripts from the CPStools pipeline (https://github.com/Xwb7533/CPStools/). Relative synonymous codon usage (RSCU) values and amino acid frequencies were calculated with default parameters. The RSCU values were plotted and visualized using an R script.

### Phylogenetic analysis

The phylogenetic relationships of *L. maroccana* within its genus and family were analyzed using complete chloroplast genome sequences and selected gene regions. Complete chloroplast genomes of 12 closely related *L. maroccana* species were retrived from NCBI and aligned using MAFFT [41] with the *--auto* option for optimal alignment strategy. The resulting multiple sequence alignment was then trimmed using trimAl v1.2 [42] with the *--automated1* parameter to remove poorly aligned regions and gaps.

A maximum-likelihood (ML) phylogenetic tree was subsequently constructed using FastTree v2.1.11 [43] under the generalized time-reversible (GTR) model with Gamma-distributed rate heterogeneity (-gtr -gamma options). The resulting tree topology was evaluated using local support values computed via the Shimodaira–Hasegawa (SH)-like test.

Additionally, the *trn* and *trnk* gene sequences from 31 *Lavandula* landraces were retrieved from NCBI and analyzed separately, following the same alignment and trimming procedures as those used for phylogenetic tree construction with the complete choroplast genomes of *Lavendual* species. All phylogenetic trees were visualized and edited using Mega 6 [44].

### Chloroplast DNA insertion into the nuclear genome

Chloroplast DNA (cpDNA) transfers to the nuclear genome were identified using a BLAST-based approach. The assembled *Lavandula maroccana* chloroplast genome was compared against the nuclear genome of *Lavandula angustifolia* using stringent BLAST parameters (e-value <10 ⁻ ⁵, hit length ≥100 bp) to detect homologous sequences. Non-overlapping Blast hits were filtered using Python scripts. This approach identified potential chloroplast-derived insertions in the nuclear genome. To find the full-length Cp gene insertion in the nuclear genome, we blast all the CP genes into the chromosomes of the genome using the blast parameter with an e-value <10 ⁻ ⁵. We then filter the blast hits based on identity percentages of 95% and query coverage of 100% for the gene.

## Results

### Genome structure and features

**Fig 1:**
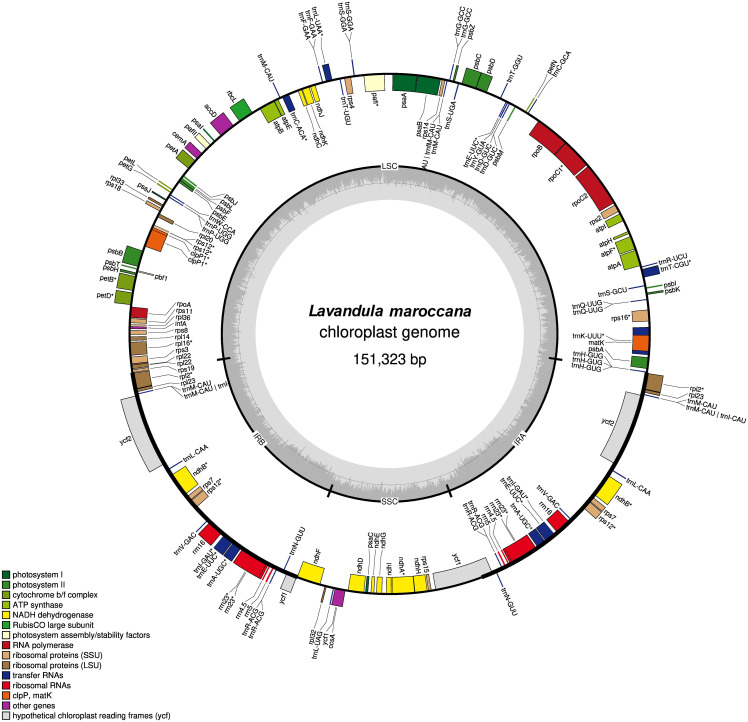
Map of the *Lavandula maroccana* chloroplast genome. Genes located inside the circle are transcribed clockwise, while those outside are transcribed counterclockwise. Genes from different functional groups are shown in various colors. The thick lines indicate the inverted repeats (IRa and IRb), which divide the genome into small single copy (SSC) and large single copy (LSC) regions.

The complete chloroplast genome of *L. maroccana* was successfully assembled and annotated using 23M reads totalling 3.3 GB of whole-genome short-read sequencing data. The genome exhibits a typical circular quadripartite structure (Fig 1) with a total length of 151,323 bp. It comprises a large single-copy (LSC) region of 82,861 bp, a small single-copy (SSC) region of 17,452 bp, and two inverted repeat (IR) regions, each spanning 25,505 bp (Table 1).

**Table 1:** Comparative chloroplast genome features of the *Lavandula maroccana* and its relatives.

Genotypes	Genome size (bp)	LSC size (bp)	SSC size (bp)	IR size (bp)	Total No of Gene	Unique gene number	rRNA Gene	tRNA Gene	GC%
*L. maroccana*	151323	82861	17452	25505	149	110	4	37	38
*L. angustifolia* Shansi	153448	84588	17596	25632	153	109	4	37	38
*L. angustifolia* jingxun2	151459	82945	17580	24467	149	110	4	37	38
*L. angustifolia* BPTPS143	151479	82738	17579	25581	149	110	4	37	38
*L. angustifolia* BPTPS104	151434	82682	17580	25586	149	110	4	37	38
*L. dentata*	151696	82897	17583	25608	149	110	4	37	38

In total, 149 genes were annotated in the chloroplast genome, including 110 unique genes. These include 4 ribosomal RNA (rRNA) genes and 37 transfer RNA (tRNA) genes. The GC content of the chloroplast genome was calculated at 38%. Most of the genes in the *L. maroccana* chloroplast genome were found as single copies. However, several genes were identified in multiple copies, primarily due to their localization within the inverted repeat (IR) regions. These include all four ribosomal RNA (rRNA) genes (*rrn16*, *rrn23*, *rrn4.5*, and *rrn5*), *ndhB*, *clpP1*, and the majority of the transfer RNA (tRNA) genes (S1 Table).

A total of 20 genes were identified with introns and exons (S2 Table), with exon numbers ranging from two to three, except for the intronless *trnV-UAC*. Exon lengths varied from 6 bp (*petB*) to 2612 bp (*rrn23*), while intron lengths ranged from 497 bp (*trnL*) to 2511 bp (*trnK*). Particularly, *clpP1, pafI,* and *rrn23* each contained two introns, whereas the remaining genes possessed a single intron. The *rps12* gene showed a distinct trans-spliced structure. Furthermore, the large intron in *trnK* may function as a transcription hotspot for non-coding RNAs.

**Fig 2:**
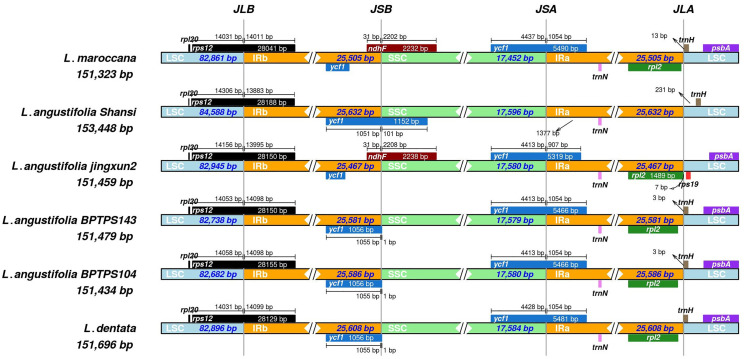
Comparison of the LSC, IR, and SSC border regions among the *Lavandula maroccana* chloroplast genomes and other *Lavandula* spp. JLB (IRb/LSC), JSB (IRb/SSC), JSA (SSC/IRa) and JLA (IRa/LSC).

The boundaries of the LSC, IR, and SSC regions were compared across the chloroplast genomes of six *Lavandula* species (Fig 2), revealing both conserved and variable features. At the JLB (IRb/LSC) region, all six species exhibited a highly conserved structure, with the *rps12* gene consistently located at this junction. In the JSB (IRb/SSC) region, both the *ycf1* and *ndhF* genes were identified; however, the length of the *ycf1* gene varied among the species. Interestingly, *L. maroccana* and *L. angustifolia* (Jingxun2) contained the *ndhF* gene in this region, while it was absent in other species. The JSA (SSC/IRa) region was largely conserved across the species, except in *L. angustifolia* (Shansi), which lacked the *ycf1* gene at this junction. In the JLA (IRa/LSC) region, the *rpl2* and *trnH* genes were consistently present across all species analyzed. These findings highlight a high degree of conservation in the IR boundary regions among the studied *Lavandula* species, with specific variations in the JSB and JSA regions suggesting possible evolutionary adaptations or genomic reorganizations.

### Sequence divergence analysis

**Fig 3:**
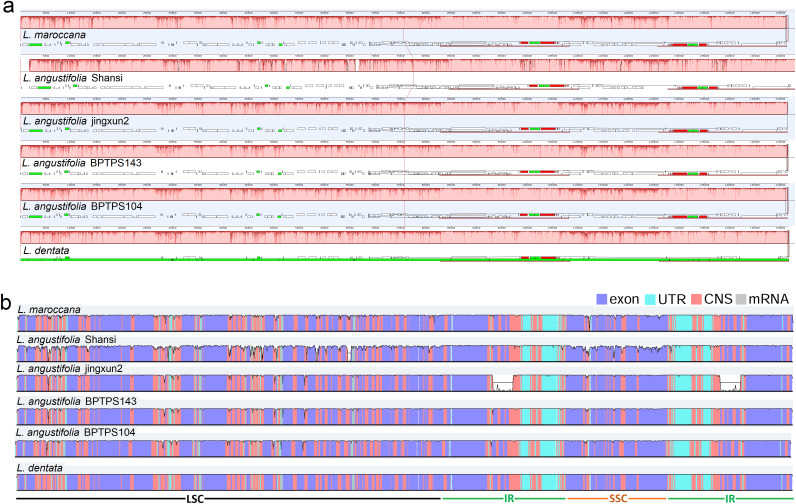
Comparative sequence analysis of complete chloroplast genomes: (a) Mauve alignment of six lavender chloroplast genomes, showing no interspecific rearrangements. (b) mVISTA-based similarity graph of six lavender chloroplast genomes, with *L. dentata* used as the reference.

The Mauve alignment of the cp genome of *L. maroccana* with other five *Lavandula* species revealed a high degree of structural conservation. No inter- or intraspecific rearrangements were identified among the genomes analyzed (Fig 3a). The overall structure and gene order were highly similar across all species, indicating strong conservation of the quadripartite structure characteristic of chloroplast genomes. To investigate sequence divergence further, a mVISTA analysis was conducted, and the results are presented in Fig 3b. The analysis confirmed a high sequence identity among the chloroplast genomes of *Lavandula* species, consistent with the findings from the Mauve alignment. Divergent regions were observed, particularly in the Large Single Copy (LSC) and Small Single Copy (SSC) regions, which were less conserved compared to the highly conserved Inverted Repeat (IR) regions. Despite the high degree of synteny and conserved gene order across the genomes, specific structural variations were identified. Notably, deletions in the IR regions were detected in the *rps12* and *ndhB* genes of *L. angustifolia* Jingxun2. These deletions represent potential markers for species-specific differentiation within the genus and may provide insights into the evolutionary dynamics of chloroplast genomes in *Lavandula*.

Nucleotide diversity (Pi) is a critical metric for identifying divergence hotspots between species. We calculated Pi values for the chloroplast genes and intergenic regions of *L. maroccana*, using *L dentata* as the reference. As shown in Figs 5a and 5b, the nucleotide diversity (Pi) index ranged from 0 to 0.0088 for genes, with an average value of 0.0035, and from 0 to 0.382 for intergenic regions. The average Pi value for genic regions was 0.008, while for intergenic regions, it was 0.015 (S3 Table). Notably, the genes *atpF*, *matK*, and *rps16* exhibited Pi values greater than 0.020, indicating higher levels of nucleotide variability.

**Fig 4:**
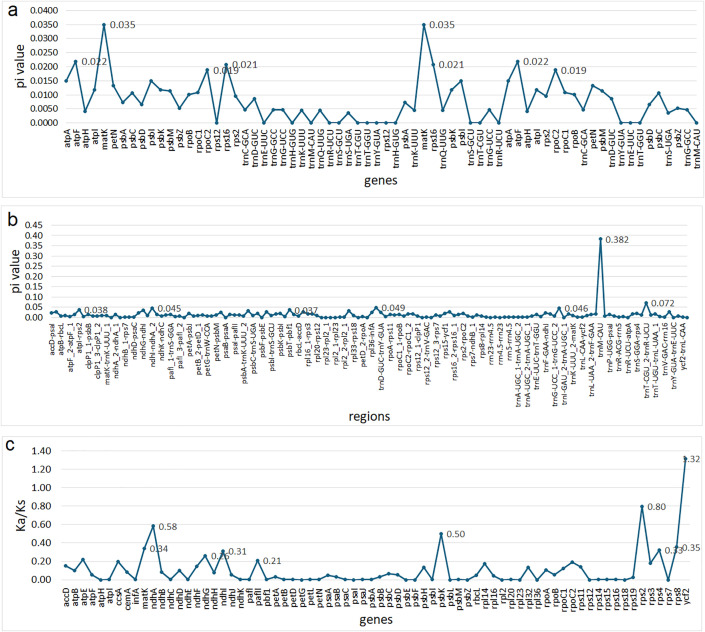
Nucleotide diversity (Pi) and Ka/Ks analysis of the chloroplast genome of *L. maroccana.* **(a)** Nucleotide diversity (Pi) across coding regions (genes). The x-axis represents the position of genes along the chloroplast genome, and the y-axis represents nucleotide diversity (Pi). **(b)** Nucleotide diversity (Pi) across non-coding regions (intergenic regions). The x-axis represents the position of intergenic regions along the chloroplast genome, and the y-axis represents nucleotide diversity (Pi). **(c)** Ka/Ks ratio analysis of protein-coding genes. The x-axis represents individual protein-coding genes, and the y-axis represents the Ka/Ks ratio.

The Ka/Ks analysis of protein-coding genes in the chloroplast genomes of *L. maroccana* species revealed a range of values from 0 to 1.32, with an average Ka/Ks ratio of 0.20. Most genes exhibited Ka/Ks ratios below the average, indicating purifying selection acting on the majority of chloroplast genes. However, a few genes displayed elevated Ka/Ks ratios, suggesting potential adaptive evolution or relaxed selection pressures. The highest Ka/Ks ratio was observed for the gene *ycf1* (Ka/Ks = 1.32), exceeding the threshold of neutrality and indicating potential positive selection. Additionally, the top five genes with notable Ka/Ks values were *rps2* (0.80), *ndhA* (0.58), *psbK* (0.50), and *rpsB* (0.35). These findings suggest that while most chloroplast genes are highly conserved, certain genes like *ycf1* and *rps2* may have undergone adaptive changes, which could be associated with functional diversification or environmental adaptation within the genus *Lavandula*.

### Analyses of repeats and simple sequence repeats (SSR)

Repeats and simple sequence repeats (SSRs) were analyzed for the *L. maroccana* chloroplast genome and compared with five other *Lavandula* species. The results are shown in Figs 4a-4f. The analysis revealed that complementary, reverse, forward, and palindromic repeats occurred at similar frequencies across most of the studied species, except for the *L. angustifolia* Shansi sample, where reverse repeats were significantly more abundant. Palindromic and forward repeats constituted the majority of repeat types in the chloroplast genomes of the *Lavandula* species analyzed, accounting for over 40% of the total repeats. In contrast, reverse and complementary repeats were less frequent, representing less than 10% of the repeats in most species. However, the *L. angustifolia* Shanssi sample was an exception, with reverse repeats exceeding 50% of the total repeat content (Fig 4a).

The analysis of simple sequence repeats (SSRs) in the chloroplast genomes of six *Lavandula* species revealed a low frequency of SSRs. It was observed that one SSR occurred approximately every 3,000–4,000 base pairs (Fig 4b), with the density ranging from 0.25 to 0.31 SSRs per kilobase. Mononucleotide repeats were dominant among both perfect and imperfect SSR types. Perfect di-, tri-, and tetranucleotide repeats were present in low numbers, typically ranging from 5 to fewer than 10 per genome. In contrast, penta- and hexanucleotide repeats were completely absent in the perfect motif category. For imperfect motifs, however, penta- and hexanucleotide repeats were identified, though their frequency was also low, with only 1–2 occurrences per genome. Most SSRs were composed of AT-rich motifs, primarily dominated by poly-A and poly-T repeats, while no GC-rich motifs were detected among mononucleotide repeats. The majority of SSRs belonged to Class II (<20 bp), indicating a preference for shorter motifs, likely to minimize replication errors and maintain genomic stability.

### Codon usage bias analysis

The protein-coding genes in *L. maroccana* collectively encode a total of 18,379 codons. The relative synonymous codon usage (RSCU) values for these codons range from 1.92 (for TTA) to 0.34 (for AGC) (S4 Table). Among the amino acids, leucine (Leu), arginine (Arg), and serine (Ser) are the most frequently encoded, reflecting their high codon usage. In contrast, methionine (Met) and tryptophan (Trp) are the least frequently represented amino acids, as each is encoded by a single codon. This distribution of codon usage and amino acid frequency highlights potential evolutionary adaptations in the chloroplast genome of *L. maroccana* (Fig 6).

### Genetic diversity and phylogenetic relationships

We constructed two phylogenetic analyses (Fig 7), one based on *trnK-matK* gene sequences and another using whole chloroplast genomes. The first phylogenetic analysis was performed using the maximum likelihood (ML) method with 15 whole chloroplast genomes of *Lavandula* and its closely related species. The analysis revealed that *L. maroccana* clustered with other *Lavandula* species, forming a distinct clade. Other species grouped according to their respective genera. The ML tree also showed that the two *Ocimum* species, the two *Mentha* species, and the two *Scutellaria* species clustered together in their respective clades. This analysis highlights the genetic diversity among closely related species and provides valuable insights into their evolutionary relationships. The second phylogenetic analysis was done using *matK* and *trnK* gene sequences of 31 taxa with the ML method provides valuable insights into the evolutionary relationships within the genus *Lavandula*, particularly at the sectional level and the placement of *L. maroccana*. The analysis highlights phylogenetic relationships among subgenera, sections, and endemic species from North Africa (*L. maroccana*), Macaronesia, and Arabia. The 31 taxa are divided into five well-supported clades: Pterostoechas, Subnudae, Stoechas, Angustifolia, and Dentatae, showing genetic divergence and taxonomic affinities.

The Pterostoechas clade, with moderate to high bootstrap support (86%), includes *L. tenuisecta*, *L. mairei*, *L. redjalii*, and *L. maroccana*. The placement of *L. maroccana* (bootstrap value 62%) suggests a close evolutionary relationship with other North African species, supporting its classification within the Pterostoechas section. The Subnudae clade, strongly supported (94%), comprises *L. buchii*, *L. multifida*, and *L. canariensis*. These species, primarily distributed in the Canary Islands and adjacent regions, demonstrate geographic and evolutionary distinctiveness. Close genetic relationships among *L. buchii*, *L. canariensis*, and *L. buchii var. gracilis* suggest recent divergence. The Stoechas clade, robustly supported (99%), includes *L. stoechas subsp. stoechas*, *L. pedunculata subsp. pedunculata*, and *L. dhofarensis subsp. dhofarensis*. Characterized by dense inflorescences and bracts, this group is adapted to Mediterranean and Middle Eastern climates, with phylogenetic clustering affirming the genetic coherence of the Stoechas section. The Dentatae subgroup, with moderate support (33%), includes *L. dentata* and *L. heterophylla*, characterized morphologically by toothed leaves. The Angustifolia subgroup, with high support (91%), comprises *L. angustifolia*, *L. intermedia*, and *L. lanata*. The inclusion of hybrids like *L. intermedia* highlights its derivation from *L. angustifolia* and *L. latifolia*. Members of this subgroup, widely distributed in temperate regions, are economically significant for essential oil production.

### Chloroplast DNA integration into the nuclear genome of Lavandula

A total of 412 BLAST hits greater than 100 bp in length, corresponding to chloroplast (cp) DNA insertions, were identified in the nuclear genome of *Lavandula angustifolia*, spanning 112,637 bp of the cp genome. This represents 74% of the total cp genome integrated into the nuclear genome. These insertions were distributed across all 25 chromosomes, with varying frequencies and alignment lengths. The proportion of cp insertions ranged from 1.62% (chr01, 1,821 bp) to 9.82% (chr09, 11,057 bp), indicating differential levels of cpDNA retention among chromosomes. Chromosome 3 exhibited the highest number of BLAST hits [30], while chromosome 24 had the lowest [7]. The average cpDNA integration length per chromosome was 4,505 bp, covering approximately 4% of all cp insertions in the nuclear genome (Table 2 and Fig 8).

**Table 2:** Summary of chloroplast DNA Integration into the nuclear genome of lavandula.

Chromosome	Blast hit Count	CP genome alignment length	Proportion of (%) all cp insertions	Nt genome alignment length
chr01	11	1821	1.62	1806
chr02	12	2937	2.61	2942
chr03	30	7527	6.68	7462
chr04	15	5179	4.60	5121
chr05	10	2398	2.13	2382
chr06	19	3615	3.21	3615
chr07	26	6002	5.33	5987
chr08	15	5609	4.98	5581
chr09	20	11057	9.82	11042
chr10	18	3398	3.02	3365
chr11	19	6721	5.97	6688
chr12	17	6260	5.56	6256
chr13	13	3337	2.96	3331
chr14	20	4339	3.85	4277
chr15	12	5098	4.53	5023
chr16	12	3896	3.46	3824
chr17	25	6052	5.37	6016
chr18	18	3866	3.43	3854
chr19	14	2979	2.64	2960
chr20	14	2548	2.26	2513
chr21	15	3473	3.08	3438
chr22	14	4631	4.11	4671
chr23	24	5456	4.84	5427
chr24	7	2511	2.23	2456
chr25	12	1927	1.71	1902
Max	30	11057	9.82	11042
Min	7	1821	1.62	1806
Average	16	4505	4.00	4478
Grand Total	412	112637	100	111939

**Fig 5:**
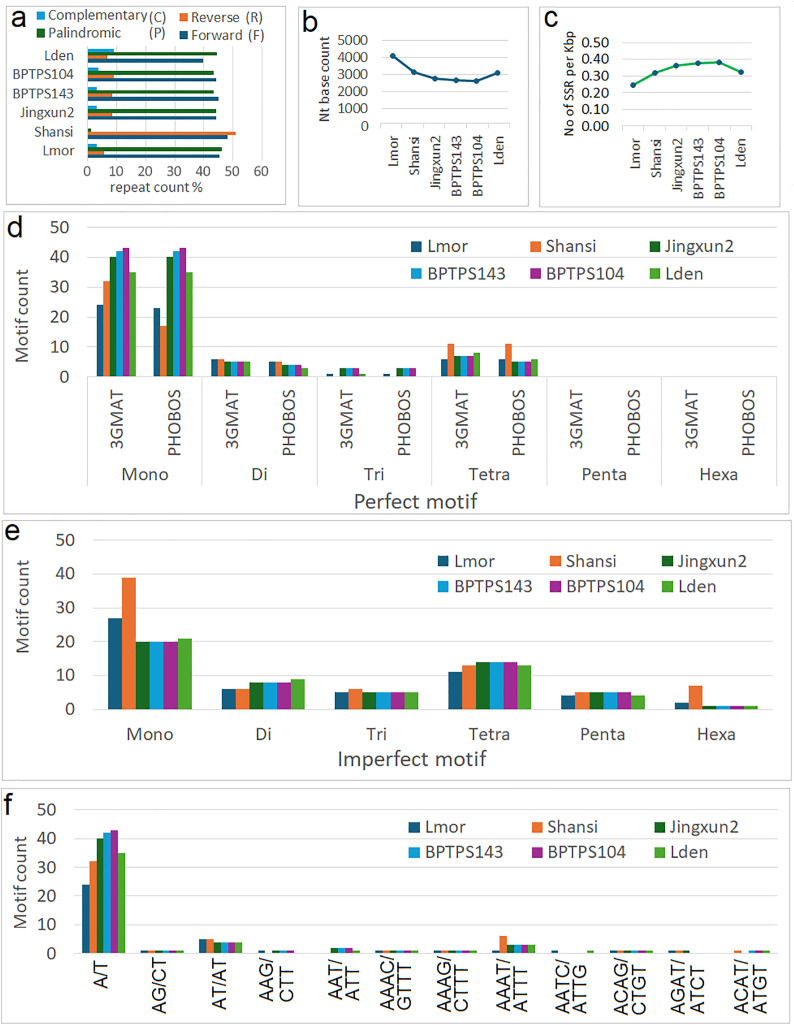
Analyses of long and short repeats: **(a)** Comparative distribution of long repetitive sequences in six complete chloroplast genomes of *Lavandula* species **(b)** SSR density of the in six complete chloroplast genomes of *Lavandula* species (c) no of SSR found per kbp cp genomes (d) distributions of perfect SSR motifs among in six complete chloroplast genomes of *Lavandula* species compare with both tools result 3GMAT and PHOBOS (e) distribution of imperfect motif of SSR among in six complete chloroplast genomes of *Lavandula* species (f) comparative distributions of motif types.

**Fig 6:**
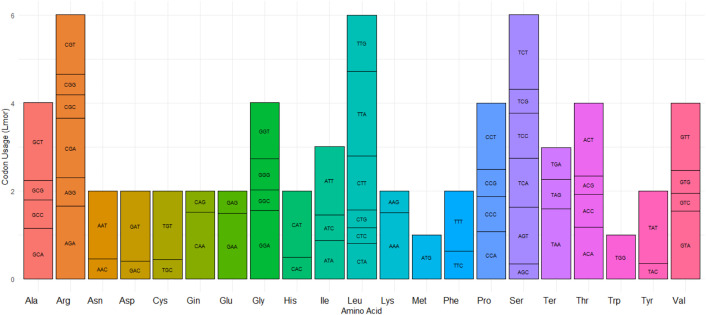
Codon usage of 20 amino acids and stop codons in protein-coding genes of the *L. maroccana* chloroplast genome.

**Fig 7:**
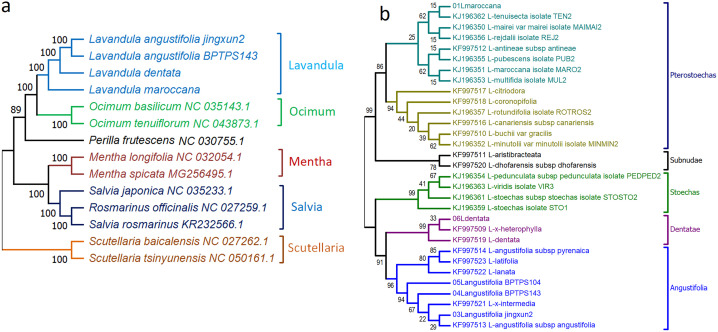
Phylogenetic tree showing the evolutionary relationships of *Lavandula* species and their closely related genera, constructed using the maximum likelihood (ML) method. Panel (a) represents the tree based on 15 whole chloroplast genomes, with *Arabidopsis thaliana* used as the outgroup. Panel (b) shows the tree constructed from *trnK-matK* gene sequences of 31 *Lavandula* species. Bootstrap values greater than 50% are indicated at the branches.

**Fig 8:**
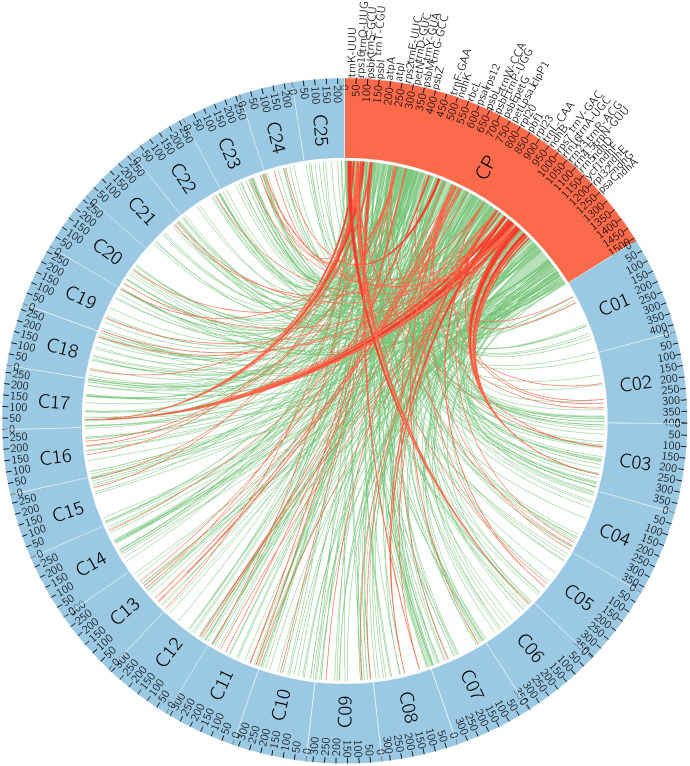
The Circos plot represents the insertion of the *L. maroccana* chloroplast genome and full-length CP gene insertions in the nuclear genome of *L. angustifolia* across 25 chromosomes. The CP genome is shown on a bp scale, while the chromosomes are represented on a Kb scale. Green lines indicate CP genome insertions into the nuclear genome, whereas full-length CP gene insertions are highlighted in red.

A total of 53 chloroplast genes were identified as full-length insertions in the nuclear genome, with a total of 104 copies (S5 Table). The copy number varied from 1 to 7. Among them, the transfer RNA gene *trnV-GAC* exhibited the highest copy number (7), followed by *trnL-CAA* (6), *psbI* (5), and *trnN-GUU* (5). Several genes, including *rrn4.5*, *rrn5*, *trnS-GCU*, and *trnQ-UUG*, were found in 3–4 copies. The gene *ycf1*, known for its role in chloroplast function and genome stability, was detected in two copies.

## Discussion

The complete chloroplast genome plays a key role in understanding the evolutionary relationships, phylogenetics [45–48], and plant adaptation [49] due to its conserved and moderately variable structure. The complete chloroplast genome of *L. maroccana,* an endangered species listed in the IUCN red list in 2020 [5], was successfully sequenced, assembled and annotated using short-read whole-genome sequencing data. Similar methods have been effectively applied to numerous plant species, such as *Chrysanthemum morifolium* [50], *Juniperus* spp. [51], and *Neltuma pallida* [52], as a reliable approach for chloroplast genome studies. Comparative genomic analysis revealed that the assembled *L. maroccana* chloroplast genome is similar in length and structure to those of other *Lavandula* species analysed in our study, including *L. dentata* and *L. angustifolia*. The total genome length, gene count, and structural organization of the *L. maroccana* chloroplast genome closely resemble to other *Lavandula* species, reflecting a conserved genomic architecture within the genus. Chloroplast genome conservation within plant genera is a well-known, characterized by similarities in gene content, order, and overall structure. This high level of conservation reported in genera, such as *Arabidopsis* [53], *Oryza* [54], *Avena* [17] and *Pinus* [55] can be attributed to the essential roles of chloroplast genes in photosynthesis and metabolism, as well as the evolutionary constraints associated with these functions.

A comparative analysis of the inverted repeat (IR) boundaries across six *Lavandula* species revealed both conserved and variable features. The consistent IR boundary structure, including the positioning of genes such as *rps12* and *ycf1*, highlights the structural stability of the *Lavandula* chloroplast genome. Similar features also reported across Lamiaceae and other eudicot lineages for example *Salvia* species, the *rps19* gene typically spans the LSC/IRb boundary, producing a truncated *ψrps19* in IRa, while *ycf1* commonly crosses the SSC/IRa boundary, forming a large pseudogene fragment in the adjacent IR region, mirroring the arrangement in *Lavandula* [56,57]. However, variations in the length and presence of specific genes, such as *ndhF* and *ycf1*, particularly in the JSB and JSA regions, suggest potential evolutionary adaptations to ecological pressures or genomic reorganization events. These variations provide valuable insights into the phylogenetic dynamics within the genus *Lavandula*.

Sequence divergence analysis using Mauve alignment and mVISTA confirmed the structural conservation of the *Lavandula* chloroplast genomes. The absence of major rearrangements, such as inversions or translocations, further supports the evolutionary stability of the species. Despite this, nucleotide diversity (Pi) analysis identified areas of higher divergence, particularly in the LSC and SSC regions. These regions may be under different selective pressures compared to the more conserved IR regions. Notably, genes such as *atpF*, *matK*, and *rps16* exhibited higher nucleotide variability, indicating their potential role in adaptive evolution within the genus. Similar observation also reported in other plants species such as *Salvia*, and *Jasminum* [58]. Ka/Ks analysis provided further evidence of purifying selection acting on most chloroplast genes. However, the elevated Ka/Ks ratio for the *ycf1* gene suggests it may have undergone positive selection, consistent with its critical role in chloroplast function and potential involvement in environmental adaptation. Elevated Ka/Ks ratios for other genes, such as *rps2*, *ndhA*, and *psbK*, suggest functional diversification or a relaxation of selection pressures, which may contribute to evolutionary divergence within the genus.

The analysis of simple sequence repeats (SSRs) in the *Lavandula* chloroplast genome revealed a low frequency of SSRs, consistent with the conserved and compact nature of chloroplast genomes. Across the six *Lavandula* chloroplast genomes analyzed, SSRs were predominantly AT-rich, aligning with trends observed in other plant species such as citrus [59], banana [60], and enset [61]. The prevalence of poly-A and poly-T motifs is likely due to the abundance of non-coding regions, where AT-rich sequences enhance genome stability and replication efficiency. No GC-rich SSR motifs were detected, as selective pressures generally disfavor GC-rich sequences that could compromise genome stability. Few trinucleotide repeats were observed, reflecting evolutionary constraints against such repeats in *Lavandula* chloroplast genomes, where they could disrupt protein function if located in coding regions. Longer repeats, such as penta- and hexanucleotide motifs, were rarely present, while shorter motifs were more frequent, likely due to the small genome size and purifying selection against larger repeats. Most detected SSRs were Class II (<20 bp), indicating a preference for shorter repeats to minimize replication slippage and maintain genomic integrity. Similar trends have also been observed in other *Lamiaceae* species such as *Mentha longifolia*, *Ocimum basilicum*, *Salvia miltiorrhiza*, and *Perilla frutescens* [62]. The congruence between the cpDNA-based tree and the nuclear gene-based phylogeny reinforces the evolutionary placement of *Lavandula* within *Lamiaceae*. While the chloroplast genome provides reliable resolution at the genus level, the nuclear dataset offers finer resolution of intergeneric relationships and captures deeper evolutionary divergence across the family. These findings highlight the unique characteristics and evolutionary pressures shaping SSR distribution in the *Lavandula maroccana* chloroplast genome. They contribute to a broader understanding of chloroplast genome stability within the genus and the evolutionary forces that influence genome organization and adaptation.

Phylogenetic analyses using whole chloroplast genomes and *trnK-matK* sequences provide complementary insights into the evolution of *Lavandula* species. The chloroplast genome-based ML tree clusters *L. maroccana* with other *Lavandula* species, confirming its genetic affiliation within the genus, while other genera (*Ocimum*, *Mentha*, *Scutellaria*) form distinct clades. The *trnK-matK*-based ML tree resolves *Lavandula* into five clades: Pterostoechas, Subnudae, Stoechas, Angustifolia, and Dentatae. *L. maroccana* groups within the Pterostoechas clade (bootstrap 62%), emphasizing its evolutionary link to North African species and adaptation to arid environments. Strong support for Subnudae (94%) and Stoechas (99%) confirms their distinctiveness, while the Angustifolia clade (91%) includes hybrids like *L. intermedia*, highlighting its economic importance. This clade’s adaptation to arid and semi-arid environments reflects ecological specialization.

These analyses align molecular evidence with traditional classifications, showcasing genetic diversity, ecological adaptation, and taxonomic clarity within *Lavandula*. Our findings are consistent with previous studies [63], which confirmed the monophyly of *Lavandula* and delineated its subgenera and sections. The identification of five well-supported clades—Pterostoechas, Subnudae, Stoechas, Angustifolia, and Dentatae—reinforces the robustness of our results and contributes to a more comprehensive understanding of *Lavandula* phylogeny, with implications for conservation and breeding.

The integration of chloroplast DNA (cpDNA) into the nuclear genome is a well-documented phenomenon in plant evolution, contributing to genetic diversity and genomic complexity [64]. In Lavandula angustifolia, we observed that 74% of the chloroplast genome has been integrated into the nuclear genome, with these sequences distributed across all 25 chromosomes at varying frequencies. Huang et al. reported that approximately 95% of the *Arabidopsis thaliana* chloroplast genome has been transferred to its nuclear genome [65]. Similarly, 95% integration has been documented in rice (*Oryza sativa*) [66] and 90–95% in banana (*Musa spp*.) [67], while much lower levels of integration (~14%) have been reported in tobacco (*Nicotiana tabacum*) [68].

Overall, the chloroplast genome of *L. maroccana* exhibits a stable and highly conserved structure, with certain genes showing variability that may indicate evolutionary adaptation. This study provides valuable insights into the genomic characteristics of *L. maroccana* and enhances our understanding of chloroplast genome evolution within the genus *Lavandula*. Further research into the functional implications of the identified structural variations and sequence divergences will deepen our knowledge of the evolutionary mechanisms driving genetic diversity in this important plant genus.

## Supporting information

S1 Table Gene annotated in the *L. moraccona* chloroplast genome. (XLSX)

S2 Table Exon and intron count and lengths of genes in the chloroplast genome of *L. maroccana.* (XLSX)

S3 Table Pi value of the Genes and intergenic regions. (XLSX)

S4 Table Codon usage table for six Lavandula species. (XLSX)

S5 Table List of chloroplast genes found in full length in the nuclear genome of *Lavandula angustifolia.* (XLSX)
